# Room-temperature hyperpolarization *via* polarization relay through rapid cocrystallization

**DOI:** 10.1039/d6sc03071h

**Published:** 2026-07-01

**Authors:** Haruki Sato, Makoto Negoro, Akinori Kagawa, Takuya Kurihara, Ken-ichi Otake, Koichi Nakamura, Munehiro Inukai

**Affiliations:** a Graduate School of Science and Technology for Innovation, Tokushima University Tokushima 770-8506 Japan; b Center for Quantum Information and Quantum Biology, The University of Osaka Toyonaka Osaka 560-8531 Japan; c Premium Research Institute for Human Metaverse Medicine, The University of Osaka Toyonaka Osaka 560-8531 Japan; d Institute for Quantum Life Science, National Institutes for Quantum and Radiological Science and Technology Inage-Ku Chiba 263-8555 Japan; e Department of Chemistry, Graduate School of Natural Science and Technology, Kanazawa University Kanazawa Ishikawa 920-1192 Japan; f Institute for Integrated Cell-Material Sciences, Kyoto University Institute for Advanced Study, Kyoto University Kyoto 606-8501 Japan; g Graduate School of Technology, Industrial and Social Sciences, Tokushima University Tokushima 770-8506 Japan inukai.munehiro@tokushima-u.ac.jp

## Abstract

Hyperpolarization of solids at room temperature using photoexcited triplet electrons [triplet dynamic nuclear polarization (DNP)] has been limited to a small number of solid-state systems, primarily those composed of molecules with exceptionally long nuclear spin–lattice relaxation times (*T*_1_). Here, we introduce HYCOR (HYperpolarization *via* COcrystallization and Relay), which is a polarization-relay strategy that enables the transfer of hyperpolarization generated by triplet DNP from a polarized mediator to target molecules through rapid cocrystallization. As a proof of concept, we demonstrate room-temperature ^13^C polarization of pyruvic acid (PyA), a benchmark molecule in hyperpolarization studies. In this approach, polycrystalline picolinamide (PAm) containing dispersed pentacene is hyperpolarized by triplet DNP, followed by rapid mixing with liquid PyA to induce cocrystallization. The resulting PyA–PAm cocrystals exhibit ^13^C polarization of up to 0.012% in the solid state and 0.0028% after dissolution at room temperature. Analysis of the polarization loss reveals that the efficiency of HYCOR is governed by a kinetic competition between cocrystallization and *T*_1_ relaxation. HYCOR provides a concept for extending triplet DNP to target molecules that are difficult to polarize directly.

## Abstract 2

Rapid cocrystallization relays room-temperature triplet-DNP polarization from a solid mediator to pyruvic acid, expanding the scope of triplet-DNP hyperpolarization.

## Graphical abstract

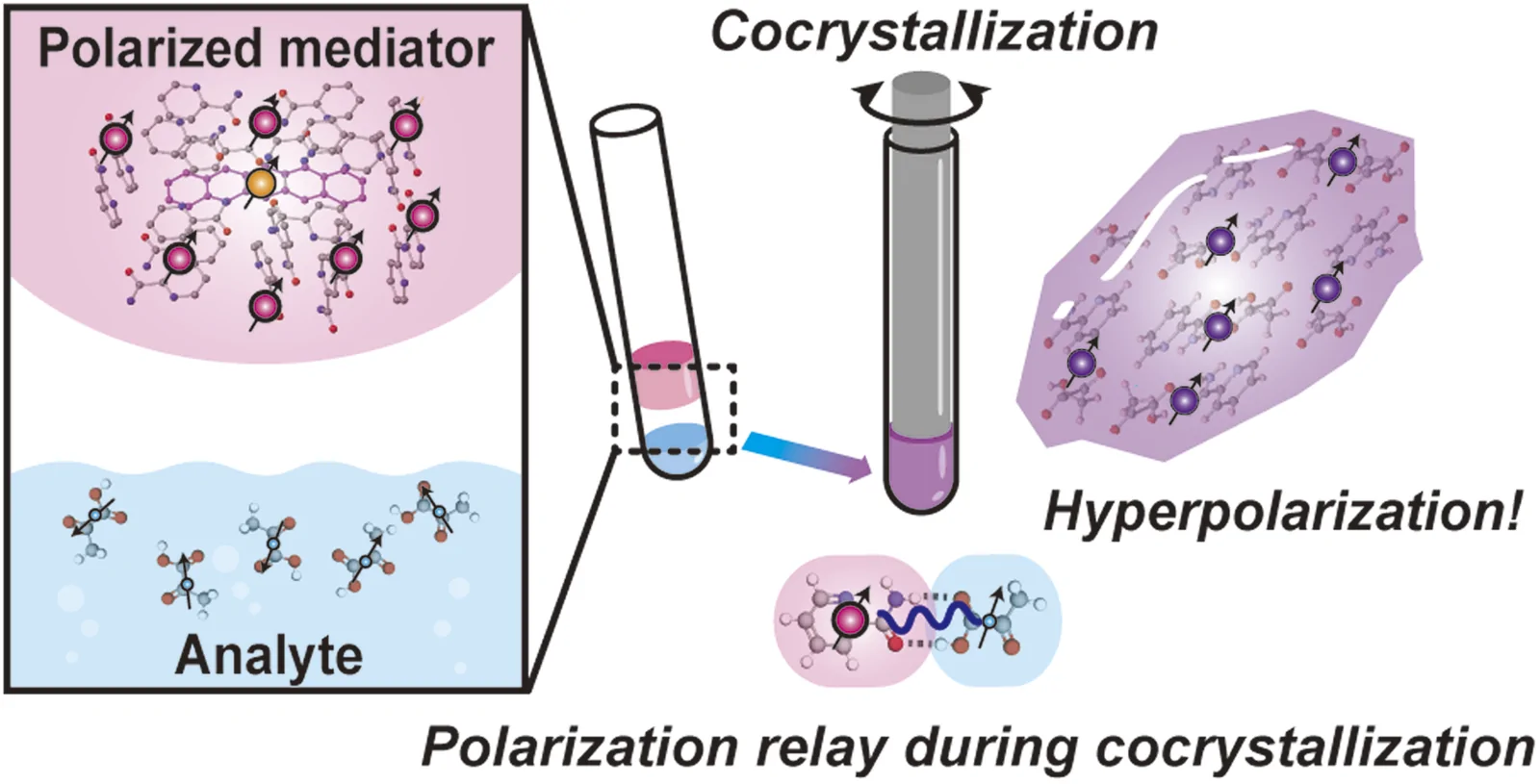

## Introduction

Nuclear magnetic resonance (NMR) is a powerful technique for probing the local structures and dynamics of molecules. However, its inherently low sensitivity often hampers applications in chemistry and life sciences. Cryogenic-radical dynamic nuclear polarization (DNP) has emerged as a promising strategy for overcoming this limitation, by transferring spin polarization from electrons to nuclei, thereby enhancing NMR signals by several orders of magnitude.^1–5^ In particular, dissolution DNP enables the transfer of polarization from the solid state to the solution state, allowing its use in solution-state NMR and MRI applications.^6–8^ However, its reliance on liquid-nitrogen or -helium cooling and millimeterwave irradiation limits its accessibility and routine use.

Several room-temperature hyperpolarization methods have been developed to overcome these limitations, including parahydrogen-induced polarization (PHIP),^9–12^ Overhauser DNP (ODNP),^1,2,13–15^ photochemically induced DNP (photo-CIDNP),^16–19^ and triplet DNP.^20–22^ These approaches differ in how polarization is delivered to the target molecule, such as through chemical exchange, interaction with paramagnetic species, or photoinduced processes. However, each method requires specific chemical or physical conditions, which restricts its applicability across diverse molecular systems.

In particular, triplet DNP, which exploits the high spin polarization of photoexcited triplet states, can generate highly polarized solids at room temperature and in low magnetic fields. However, efficient triplet DNP imposes two critical requirements for the solids: the homogeneous dispersion of the polarization source and exceptionally long nuclear spin–lattice relaxation times (*T*_1_) at room temperature.^23^ These are required because triplet DNP relies on repeated pulsed-laser excitation (typically 50–1000 Hz) of dispersed polarizing agents, leading to the gradual accumulation of polarization.^24^ In recent years, considerable efforts have been devoted to developing advanced polarizing agents with improved chemical stability,^25^ high polarization,^26^ and compatibility with diverse molecular environments,^27^ leading to enhanced polarization performance under a broader range of conditions. However, despite these advances, efficient triplet DNP still requires the simultaneous fulfillment of homogeneous dispersion and sufficiently long *T*_1_, which remains challenging to realize across diverse molecular systems.

One strategy for overcoming this *T*_1_ limitation is to prepare a polarizing mediator with an inherently long *T*_1_, where hyperpolarization is first accumulated and then relayed to the target molecule. Several polarization-relay strategies—including signal amplification by reversible exchange (SABRE)^28,29^ and the combination of dissolution triplet DNP with the nuclear Overhauser effect (NOE)^30^—have been developed, in which analyte molecules are brought into proximity with the polarized species in the liquid state. In contrast, solid-state polarization relay—such as surface-enhanced DNP–NMR^31,32^—relies on the propagation of spin polarization *via* spin diffusion within the nuclear relaxation time scale, and therefore requires that target spins be located within the spin-diffusion range of the polarization source. This constraint becomes critical in heterogeneous or phase-separated solids, or when the polarization source cannot be uniformly distributed in close proximity to the target molecules.

**Scheme 1:**
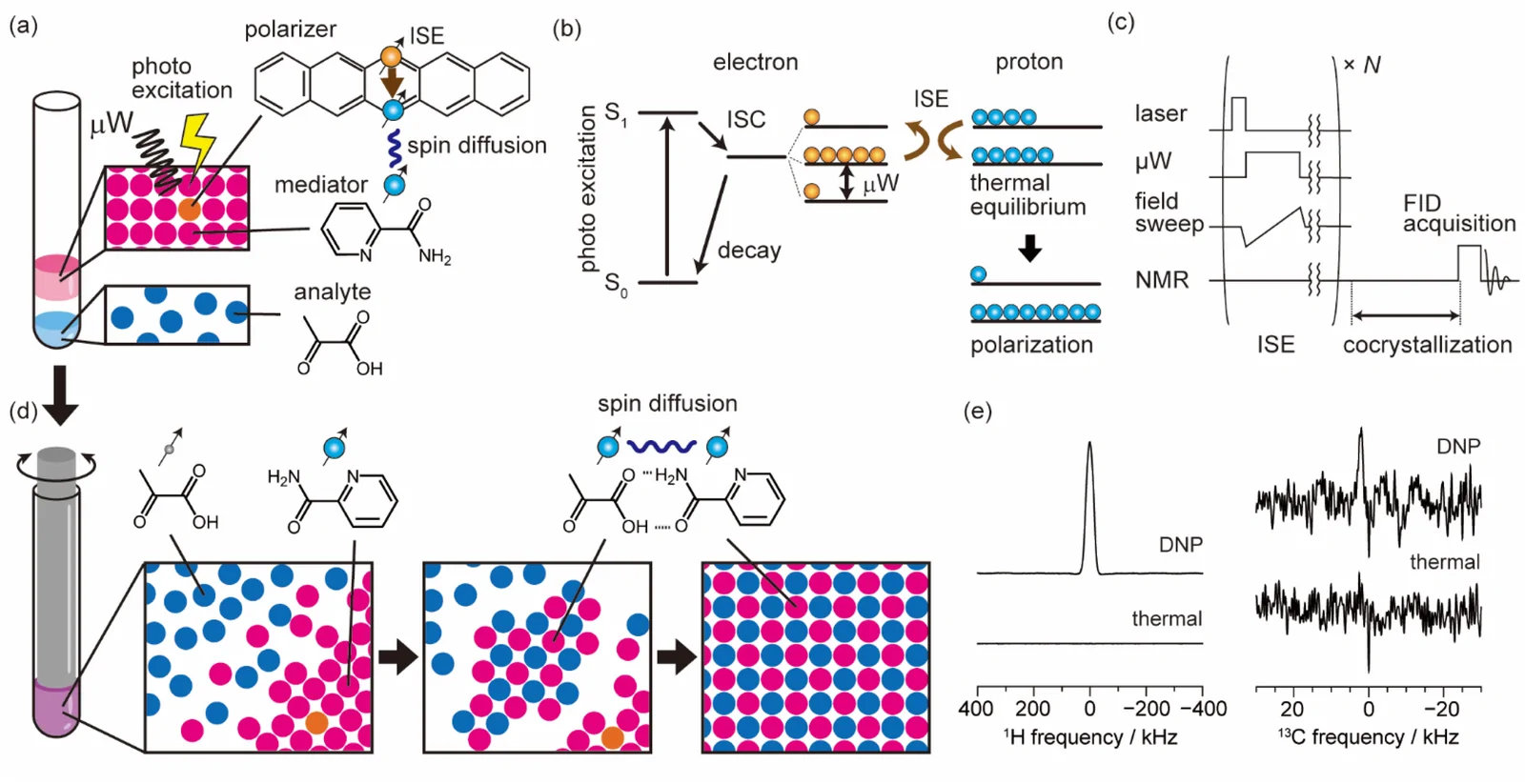
Concept of polarization relay *via* rapid cocrystallization. (a) Triplet DNP of a solid relay mediator (*e.g.*, picolinamide containing dispersed pentacene as the polarizer) using photoexcited triplet electrons. (b) Schematic illustration of ^1^H hyperpolarization by triplet DNP. Photoexcited triplet electrons generated in pentacene transfer their spin polarization to nearby ^1^H nuclei through the ISE and subsequently diffuse through the solid *via*^1^H–^1^H spin diffusion. (c) Experimental sequence for triplet DNP and HYCOR (HYperpolarization *via* COcystallization and Relay). (d) Rapid cocrystallization of the liquid target analyte (*e.g.*, pyruvic acid) with the hyperpolarized solid mediator inside a magnet. The polarization is relayed *via* spin diffusion from the mediator to the analyte during cocrystallization. (e) Demonstration of polarization relay *via* HYCOR (left: ^1^H NMR spectrum after 5 s cocrystallization; right: ^13^C spectrum after subsequent polarization transfer *via* CP). Triplet DNP was conducted at 0.39 T and 298 K, and the spectra were compared with the corresponding thermal spectra.

Herein, we introduce polarization relay *via* rapid cocrystallization, which we term HYCOR (HYperpolarization *via* COcystallization and Relay). In this method, nuclear spin polarization generated in a hyperpolarized mediator is transferred to target molecules during cocrystallization within a magnetic field (Scheme 1). Cocrystals are well established in crystal engineering as functional molecular assemblies formed through hydrogen-bond networks.^33–35^ Liquid-assisted cocrystallization and related solid–liquid transformations can accelerate cocrystal formation,^36^ thereby facilitating polarization relay before significant *T*_1_ relaxation of polarization occurs. Thus, the feasibility of HYCOR is governed by a kinetic competition between cocrystallization and *T*_1_ relaxation, together with the ability of the mediator and analyte to form stable cocrystals. As a proof of concept, we demonstrate room-temperature ^13^C polarization of pyruvic acid (PyA), providing a model system to examine polarization transfer during dynamic cocrystallization.

## Results and discussion

### Structure of the cocrystal

PyA was selected as the target analyte because it is one of the most widely studied molecular probes in DNP–MRI and serves as a benchmark compound for hyperpolarization methodologies.^37–39^ PAm was selected as the mediator owing to its exceptionally long *T*_1_ and high triplet-DNP polarization in hydrogen-bonded molecular solids at room temperature.^40^ The structure of the PyA–PAm cocrystal was determined by single-crystal X-ray diffraction (Table S1). A cocrystal composed of PyA and PAm is shown in Fig. S1. Direct pentacene doping into the PyA–PAm cocrystal was not achieved under the present conditions. The UV-vis spectrum showed negligible pentacene absorption (Fig. S2). In the crystal structure, PyA and PAm are connected through acid–amide hydrogen bonds between the carboxyl group of PyA and the amide group of PAm. This hydrogen-bonded network places the mediator and analyte in close proximity, enabling polarization transfer *via* spin diffusion. Additionally, PAm molecules are closely stacked *via* π–π interactions, which stabilize the molecular packing and contribute to long *T*_1_ values for both ^1^H and ^13^C, with *T*_1_ (^1^H) = 9.6–39 s and *T*_1_ (^13^C, COOH) = 5.0 × 10^1^ to 1.4 × 10^3^ s over the magnetic-field range 0.39–14.1 T (Fig. S3).

#### Rapid cocrystallization of PyA and PAm

**Fig. 1:**
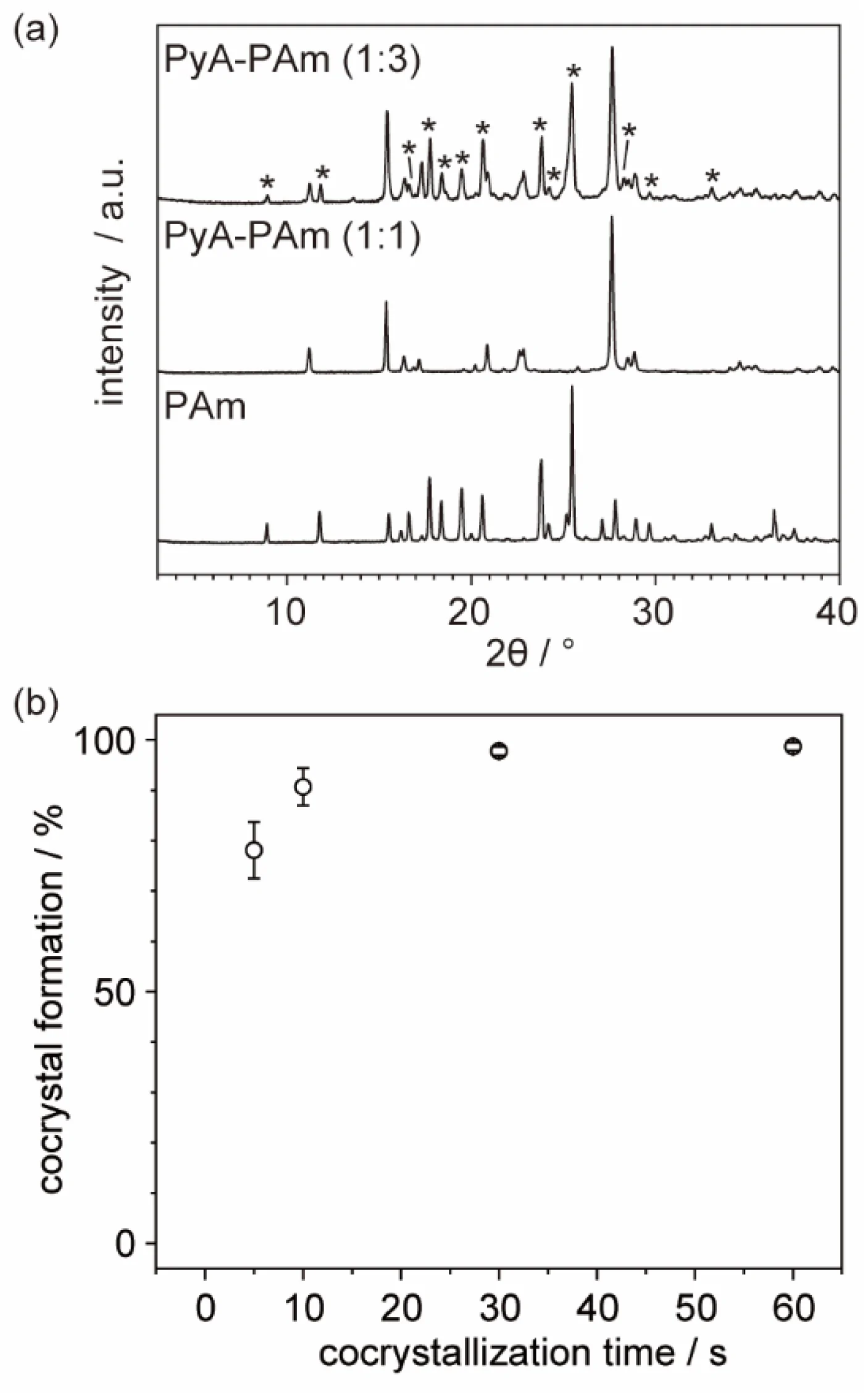
Rapid cocrystallization of PyA and PAm. (a) Powder X-ray diffraction (PXRD) patterns of PAm, the PyA–PAm cocrystal (PyA : PAm = 1 : 1), and the mixture obtained after 5 s of mixing (PyA : PAm = 1 : 3). The mixture shows contributions from both the cocrystal and unreacted PAm. Asterisks indicate peaks assigned to unreacted PAm. (b) Time evolution of the cocrystal fraction determined by PXRD using the internal-standard method.

We mixed pelletized PAm with liquid PyA at the molar ratios 1 : 1, 1 : 2, and 1 : 3 (PyA : PAm). In each case, we allowed the mixture to react until a dry crystalline powder formed. The required reaction times were approximately 120 s, 60 s, and 5 s for the 1 : 1, 1 : 2, and 1 : 3 ratios, respectively. Powder X-ray diffraction (PXRD) analysis of the sample obtained at the 1 : 3 ratio after 5 s revealed the formation of the PyA–PAm cocrystal with unreacted PAm (Fig. 1a). Because the polarization decays due to *T*_1_ relaxation after triplet DNP, cocrystallization of the target with the polarized mediator must be completed within the *T*_1_ relaxation time to enable polarization relay. Therefore, we selected the 1 : 3 ratio, which exhibits the shortest crystallization time, for further studies.

We performed cocrystallization of PyA and PAm directly in NMR tubes under the same conditions used in our DNP and polarization-relay experiments (Fig. S4). Quantitative analyses of the peak areas in PXRD using the internal-standard method indicated that the fraction of cocrystallization increased with the reaction time, reaching 78% after 5 s (Fig. 1b and S5). These results demonstrate that the majority of the reaction occurs within a few seconds, which is shorter than the relaxation time *T*_1_ (^1^H) of the cocrystal. Such rapid cocrystallization has been reported in liquid–solid reactions, which can proceed on timescales of several seconds owing to the high dispersion of the liquid phase.^41^ Based on these results, we chose the 1 : 3 (PyA : PAm) composition with the reaction time of 5 s for further experiments. While this timescale satisfies the requirement for polarization relay, the actual efficiency may still be limited by relaxation processes occurring during cocrystallization. A small fraction of the PyA remained unreacted after 5 s; however, it did not contribute to the observed hyperpolarized NMR signals (Section S5).

#### Demonstration of HYCOR using PyA

We polarized a pelletized PAm sample using triplet DNP with a 200 Hz ISE repetition rate for 1500 s at 0.39 T and 298 K. The proton polarization was *P* (^1^H) = 0.96% and the enhancement factor was *ε* (^1^H) = 7.2 × 10^3^ (Fig. S6). *ε* are referenced to the thermal polarization at 0.39 T. We then mixed the polarized PAm with liquid [1-^13^C] PyA. After 5 s of cocrystallization, the resulting mixture (PyA–PAm cocrystals and unreacted PyA and PAm) exhibited *P* (^1^H) = 0.57% [*ε* (^1^H) = 4.3 × 10^3^] (Scheme 1e). The triplet DNP pulse sequence and HYCOR experimental timing are summarized in Scheme 1c. When the polarization was subsequently transferred to the carbon nuclei *via* cross-polarization (CP), the mixture exhibited a carbon polarization *P* (^13^C) = 0.074% [*ε* (^13^C) = 2.2 × 10^3^] (Scheme 1e). The observed ^13^C signal comprises contributions from the labeled carbonyl carbon of [1-^13^C] PyA and natural abundance ^13^C from both PyA and PAm in the cocrystal and residual phases. Because the contribution from residual unreacted PyA was negligible under the present DNP conditions, the polarization of the cocrystal was determined by subtracting the signal from unreacted PAm using the PXRD-determined composition ratio (Section S5). The resulting polarization was *P* (^1^H) = 0.018% [*ε* (^1^H) = 1.4 × 10^2^] and *P* (^13^C) = 0.012% [*ε* (^13^C) = 3.7 × 10^2^]. The lower ^13^C polarization compared to ^1^H arises in part from polarization losses during CP.

The polarization decreases substantially from 0.96% in the hyperpolarized PAm mediator to 0.018% after the HYCOR process. The observed loss can be described by two contributions, including polarization loss during formation of the PyA–PAm cocrystal and subsequent *T*_1_ relaxation of the formed cocrystal before detection. The first contribution is associated with formation of the PyA–PAm cocrystal. Upon mixing liquid PyA with the polarized PAm mediator, the sample passes through a wet solid–liquid state before the stable cocrystal is fully formed. During this stage, polarization loss may occur through increased molecular mobility, molecular rearrangement, nucleation, and incorporation into the growing cocrystal. The second contribution is the intrinsic *T*_1_ relaxation of the formed PyA–PAm cocrystal before detection. Because the *T*_1_ (^1^H) of the cocrystal is 9.6 s at 0.39 T, the relayed polarization continues to decay after cocrystallization until NMR detection.

To evaluate these two contributions quantitatively, we constructed a simple kinetic model that combines the PXRD-derived cocrystallization kinetics, an effective polarization-loss factor during cocrystal growth, and *T*_1_ relaxation after cocrystal formation (Section S6). This analysis gives *η*_growth_ ≈ 0.05–0.07, indicating that approximately 5–7% of the polarization survives the cocrystal-growth step before the stable PyA–PAm cocrystal is fully formed. The shorter *T*_1_ (^1^H) values observed in solution are consistent with accelerated relaxation under conditions of increased molecular mobility (Fig. S7). Thus, polarization loss during cocrystal formation is the dominant limitation in the present HYCOR process.

### Solution-state NMR at high magnetic field after dissolution

**Fig. 2:**
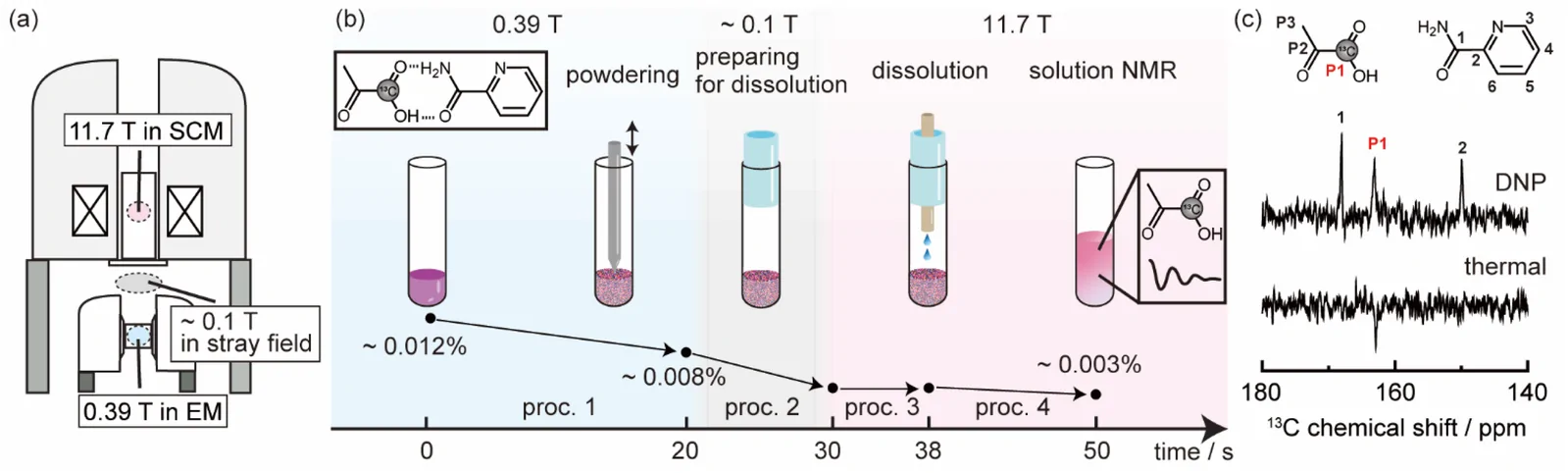
(a and b) Schematic illustration of the dissolution triplet-DNP process following HYCOR displayed along the magnetic-field and time axes. We ground the polarized PyA–PAm mixture into a powder inside an NMR tube within a 0.39 T electromagnet (EM). We attached the sample to a shuttle device in the stray field (∼0.1 T) below the 11.7 T superconducting magnet (SCM). We then transferred the polarized sample into the 11.7 T magnet, dissolved it in chloroform, and analyzed it using solution-state NMR. The approximate ^13^C polarization ratios at each stage are indicated. (c) The ^13^C NMR spectra of pyruvic acid in solution at 11.7 T after DNP and at thermal conditions. Signals from some ^13^C sites in polarized unreacted PAm (3–6) are not observed due to their rapid relaxation during dissolution, whereas sites (1, 2) with longer *T*_1_ values remain detectable (Fig. S7). Other ^13^C sites in PyA (P2 and P3) are not detected due to their low natural abundance, and signals from PAm within the cocrystal are not also observed because of both low natural abundance and the lower polarization of the cocrystal phase.

The DNP-enhanced solution-state ^13^C NMR spectrum of PyA was detected after transfer to 11.7 T and dissolution (Fig. 2). The enhanced signal appeared with a phase opposite to that of the thermal spectrum, confirming that the observed polarization originated from HYCOR. The resulting polarization was *P* (^13^C) = 0.0028%, with the enhancement factor *ε* (^13^C) = 2.8 at 11.7 T. This represents the first proof-of-concept demonstration of ^13^C polarization of PyA using polarization generated by room-temperature triplet DNP in a solid mediator. Although the polarization level remains low compared with established pyruvate hyperpolarization methods such as dissolution DNP and PHIP, the result establishes rapid cocrystallization as a pathway for relaying polarization to PyA. The dissolution process involved powdering the polarized PyA–PAm mixture at 0.39 T, transfer through the stray field (∼0.1 T), shuttling into the 11.7 T magnet, and subsequent dissolution in chloroform (Fig. 2a and b). All timing parameters were controlled and kept consistent across independent experiments.

The ^13^C polarization decreased from 0.012% to 0.0028%. This result indicates that a substantial loss of polarization occurs during the transfer and dissolution processes (Fig. 2b). We attribute this reduction to *T*_1_ relaxation processes during sample handling at 0.39 T, exposure to the stray field (∼0.1 T), and dissolution at 11.7 T (Fig. S8). At 0.39 T, where *T*_1_ (^13^C) is relatively short (50 s), the polarization decayed during the powdering step (proc. 1, 0–20 s). Additional relaxation occurred in the stray field (∼0.1 T) during sample transfer and shuttling (proc. 2, 20–30 s), where *T*_1_ is shorter (∼12 s) than at 0.39 T. In contrast, at high field (11.7 T), the solid-state PyA–PAm exhibits *T*_1_ (^13^C) of > 1000 s (Fig. S3); therefore, relaxation during this stage is negligible (proc. 3, 30–38 s). During dissolution in chloroform, an additional decay occurred within 12 s (proc. 4, 38–50 s), consistent with the liquid-state *T*_1_ (^13^C) of PyA (50 s, Fig. S9). Overall, the observed decrease in ^13^C polarization is explained quantitatively by field-dependent *T*_1_ relaxation during sample handling. This indicates that polarization loss arises from handling and transfer processes, which may be reduced by minimizing transfer time and avoiding stray field exposure. In the present proof-of-concept setup, stray-field exposure during sample transfer could not be sufficiently minimized. Further improvement may be possible by conducting sample-handling steps, such as powdering and dissolution, in the 11.7 T magnet, where *T*_1_ is longer (Fig. S3).

#### Essence and prospects of HYCOR

The concept of HYCOR addresses a key limitation of room-temperature triplet DNP, which requires an extremely long *T*_1_ for the analyte, by utilizing polarization relay from a highly polarized mediator to the analyte during cocrystallization. For HYCOR to be effective, two requirements must be satisfied: (i) the mediator and analyte must form a cocrystal, and (ii) *T*_1_ (^1^H) in the resulting cocrystal must exceed the cocrystallization time.

Because a wide variety of cocrystals have been reported and catalogued in databases such as the Cambridge Crystallographic Data Centre (CCDC), particularly in pharmaceutical research, the feasibility of cocrystal formation between mediators and analytes can be assessed. Another key direction is the exploration of diverse mediators. Expanding the mediator library directly broadens the range of cocrystal systems useful for HYCOR. In addition to picolinamide, molecules such as benzoic-acid derivatives—characterized by strong hydrogen-bonding ability, long *T*_1_, and high triplet polarization—are promising candidates. Notably, recent work has demonstrated ^1^H polarization above 60% at room temperature by triplet DNP.^42^ Furthermore, the cocrystals themselves may also function as mediators. Several hydrogen-bonded cocrystals, including 4-methylbenzoic acid–4-methylbenzamide, 4-phenylbenzoic acid–2-aminopyrimidine, and d_14_-*p*-terphenyl–1,4-di(4-pyridyl)benzene, showed room-temperature ^1^H polarization with *T*_1_ values ranging from 34 to 500 s (Fig. S10). Rapid cocrystallization of these mediator systems with small carboxylic-acid analytes such as pyruvic acid and formic acid was also observed (Fig. S10), supporting the feasibility of extending the cocrystallization step of HYCOR beyond the PyA–PAm pair.

HYCOR may also be applied to solid analytes by co-melting a DNP-hyperpolarized mediator with the analyte, and rapidly cooling the mixture to form a cocrystal, enabling spin diffusion to relay polarization to the analyte. Moreover, the HYCOR concept is not limited to triplet DNP; it may provide a general framework in which a highly polarized solid prepared using DNP acts as a mediator, facilitating polarization relay to other molecules through cocrystallization.

Despite these conceptual advantages, the present implementation of HYCOR is primarily limited by polarization loss during the formation of the PyA–PAm cocrystal, including transient wet solid–liquid contact, molecular rearrangement, nucleation, and incorporation into the growing cocrystal. The efficiency of HYCOR is governed by a kinetic competition between cocrystallization and *T*_1_ relaxation. This provides a design principle for improving polarization relay efficiency. Strategies to improve HYCOR can be classified into two directions: (i) extending *T*_1_ during cocrystal formation and (ii) accelerating cocrystallization. One effective strategy is to transfer polarization to ^13^C nuclei prior to cocrystallization, followed by ^13^C–^13^C spin diffusion within the resulting cocrystal. This approach takes advantage of the longer *T*_1_ of ^13^C nuclei, which exceeds 60 s at 1.4 T in this system (Fig. S7). Importantly, the close molecular proximity established in the cocrystal enables polarization relay even when ^13^C–^13^C spin diffusion is relatively slow. In addition, deuteration of the mediator can further suppress relaxation. Accelerating cocrystallization kinetics is also critical to ensure that polarization relay occurs before significant relaxation takes place. This can be facilitated by optimizing molecular compatibility and intermolecular interactions to promote rapid nucleation and close molecular proximity between the mediator and analyte.

## Conclusions

In this work, we demonstrated HYCOR as a polarization-relay concept that transfers polarization generated in a solid mediator to analyte molecules through rapid cocrystallization. We observed enhanced ^13^C spectra of PyA in both the solid and solution states, confirming that room-temperature triplet-DNP polarization can be relayed to PyA. Analysis of polarization attenuation indicates that the dominant loss is associated with cocrystal formation before the stable PyA–PAm cocrystal is fully formed. These results highlight the design of cocrystallization processes that preserve polarization during crystal formation as a key principle for future HYCOR systems.

## Experimental

### Preparation of the mediator

Picolinamide (PAm) doped with pentacene-d14 (0.01 mol%) was prepared by melt processing at 190 °C for 3 h, followed by rapid quenching. The resulting solid was ground and pelletized under a load of 150 kg to form cylindrical pellets.

### Triplet-DNP conditions

Triplet-DNP experiments were performed at 0.39 T and 298 K using a home-built OPENCORE NMR spectrometer^43^ equipped with a dye laser (594 nm, 6 mJ, 200 ns). The corresponding resonance frequencies were 11.6 GHz for electrons, 16.7 MHz for ^1^H, and 4.2 MHz for ^13^C. Polarization was generated using an integrated solid effect (ISE) sequence at a repetition rate of 200 Hz for 1500 s, with simultaneous microwave irradiation and magnetic-field sweep.

### HYCOR procedure

Liquid [1-^13^C] pyruvic acid (PyA) was introduced into an NMR tube, and a pellet of the mediator was placed above the liquid. The molar ratio of PyA to PAm was fixed at 1 : 3. After triplet DNP of the mediator, the two components were brought into contact, and cocrystallization was carried out for approximately 5 s in the 0.39 T electromagnet used for triplet DNP.

### NMR measurements and polarization analysis

^1^H and ^13^C NMR signals in the solid state were detected at 0.39 T. NMR detection was performed after an additional delay of approximately 2 s required for experimental setup. ^1^H signals were acquired using a magic sandwich echo sequence, and ^13^C signals were obtained *via* cross-polarization (CP) under static conditions. The ^1^H polarization was determined by comparison with thermal equilibrium signals of ethanol. The ^13^C polarization ratios were estimated using the ^1^H NMR signal intensity of ethanol at thermal equilibrium measured at 0.10 T, where the ^1^H resonance frequency matches that of ^13^C at 0.39 T.

For dissolution experiments, ^13^C NMR spectra were acquired at 11.7 T using a single-pulse sequence after sample transfer and dissolution. The ^13^C polarization in solution was determined based on the thermal equilibrium signal at 11.7 T under identical acquisition conditions. All polarization ratios were determined from three independent measurements and are reported as mean ± standard deviation (Table S2).

## Author contributions

M. Inukai conceived and supervised the project. H. Sato performed the experiments and analysed the data. M. Negoro and A. Kagawa contributed to triplet-DNP measurements and discussions. T. Kurihara contributed to solid-state NMR measurements. K. Otake performed single-crystal X-ray structure analysis. K. Nakamura contributed to data interpretation and manuscript revision. M. Inukai and H. Sato wrote the manuscript, and all authors reviewed and approved the final version.

## Conflicts of interest

There are no conflicts to declare.

## Supplementary Material

SC-017-D6SC03071H-s001

SC-017-D6SC03071H-s002

## Data Availability

The data supporting this article have been included as part of the supplementary information (SI). Supplementary information: experimental details, crystallographic data, PXRD, UV-vis, and NMR data, triplet-DNP measurements, relaxation measurements, kinetic analysis, and additional cocrystallization experiments. See DOI: https://doi.org/10.1039/d6sc03071h. CCDC 2501134 contains the supplementary crystallographic data for this paper.^44^
