# Supplementary material for: Room-temperature hyperpolarization via polarization relay through rapid cocrystallization
Source: Chem Sci. 2026 Jul 1. Online ahead of print. doi: 10.1039/d6sc03071h (PMC13344121; doi:10.1039/d6sc03071h)
Supplement: SC-OLF-D6SC03071H-s001 [file SC-OLF-D6SC03071H-s001.pdf]

Supplementary Information (SI) for Chemical Science.
This journal is © The Royal Society of Chemistry 2026

Supplementary Information for

Room-temperature hyperpolarization via polarization relay through rapid cocrystallization

Haruki Sato, Makoto Negoro, Akinori Kagawa, Takuya Kurihara, Ken-ichi Otake, Koichi Nakamura, and Munehiro Inukai

Section S1: Materials

Pyruvic acid (PyA), picolinamide (PAm), 99% [1-^{13}C] pyruvic acid ([1-^{13}C] PyA), and 95% pentacene-d14 were obtained from commercial suppliers. PAm was purified by zone-melting. Other chemicals were used without further purification. To prepare the mediator for HYCOR, PAm (10 mmol) and pentacene-d14 (0.01 mol% relative to PAm) were melted at 190°C for 3 h, and the melt was rapidly cooled using ice water for 30 s. The obtained solid was manually ground in an agate mortar for 10 s. To prepare pelletized PAm, the resulting polycrystalline powder (0.070 mmol) was pressed under a load of 150 kg for 15 s to form cylindrical pellets with a diameter of 4 mm. For HYCOR experiments, liquid PyA (0.023 mmol) was loaded into a quartz tube, and a pellet of pentacene-d14-doped PAm (0.070 mmol; PyA:PAm molar ratio = 1:3) was placed above the PyA (Figure S4).

Section S2: Physical measurements

Powder X-ray diffraction (PXRD) patterns were collected on a Rigaku Miniflex 600 diffractometer with CuKα radiation. UV-Vis measurements were carried out with a SHIMADZU UV-3600. MAS NMR measurements were performed on JEOL JNM-ECA300 and JNM-ECZ600R spectrometers at 7.0 T (MAS 5 kHz) and 14.1 T (MAS 12 kHz), respectively. Solution-state NMR experiments were performed using an 11.7 T superconducting magnet (Varian Inc.) with a home-built OPENCORE NMR spectrometer[3] and a 1.4 T benchtop NMR spectrometer (Oxford Instruments, X-Pulse). Single crystal X-ray diffraction measurements were performed at 293 K using a VariMax rotating-anode X-ray generator with confocal monochromated MoKα radiation. The structure was solved by a direct method and refined by full-matrix least-squares procedures on F2 for all reflections. The hydrogen atoms were positioned geometrically and refined using a riding model. The crystallographic data for PyA–PAm have been deposited with the Cambridge Crystallographic Data Centre (CCDC 2501134).

Section S3: Determination of cocrystal composition by PXRD (Figure S5)

The fraction of the PyA–PAm cocrystal formed under different reaction times was quantified by powder X-ray diffraction (PXRD) using an internal-standard method.[1, 2] The reaction was quenched at 5, 10, 30, and 60 s by rapidly cooling the NMR tubes in liquid nitrogen, followed by PXRD measurements of the quenched samples. The quenched samples were immediately transferred onto a PXRD sample holder pre-cooled with liquid nitrogen and subjected to PXRD measurements. No additional changes in the diffraction pattern were observed during acquisition, indicating negligible further reaction under the measurement conditions. Based on these results, the sample prepared at a PyA:PAm molar ratio of 1:3 and quenched after 5 s was selected for HYCOR experiments, as the majority of cocrystallization was completed within the T_1 (^{1}H) relaxation time of PyA–PAm.

Silicon dioxide (SiO_2) was employed as the internal standard for quantitative PXRD analysis. For calibration, pre-synthesized cocrystal was thoroughly ground with SiO_2 in an agate mortar to prepare mixtures containing 18.5, 29.2, 45.2, 55.4, and 62.3 wt% of SiO_2. PXRD patterns were collected for each mixture, and characteristic diffraction peaks of the cocrystal and SiO_2 were analyzed by profile fitting to determine their integrated peak areas. A calibration curve was constructed by plotting the integrated peak-area ratio (cocrystal / SiO_2) against the corresponding known weight ratio (cocrystal / SiO_2). The curve was used to convert the measured peak-area ratios into quantitative weight ratios. Using this calibration curve, the weight ratio of the PyA–PAm cocrystal in the mixture was determined by PXRD. The corresponding molar amount was calculated from the weight ratio using the molecular weights. For an initial feed ratio of PyA:PAm = 1:3, the resulting composition corresponds to PyA–PAm cocrystal and unreacted PAm in a molar ratio of 0.78:2.22.

Section S4: Triplet-DNP measurements

All triplet-DNP measurements were performed in a magnetic field of 0.39 T with a home-built OPENCORE NMR spectrometer and a dye laser (RDP1, Radiant Dyes). The resonant frequencies of electron, proton, and carbon were 11.6 GHz, 16.7 MHz, and 4.2 MHz, respectively. ^{1}H and ^{13}C polarized NMR signals were acquired using magic sandwich echo[4] and cross polarization after a combination of laser irradiation and an integrated solid effect (ISE) sequence.[5] The repetition rate of the ISE sequence was 200 Hz. A wavelength, energy, and pulse duration of the laser were 594 nm, 6 mJ, and 200 ns, respectively. A field-sweep width and a microwave pulse width were ± 15 mT and 25 μs, respectively. ^{1}H polarization ratio and enhancement factors were estimated using the NMR signal intensities referenced to those of ethanol at thermal equilibrium. ^{13}C polarization ratios and enhancement factors were estimated using the ^{1}H NMR

signal intensities of ethanol at thermal equilibrium measured at 0.10 T, at which the ^{1}H resonance frequency matches that of ^{13}C at 0.39 T, providing an appropriate thermal equilibrium reference. The polarization ratios were determined from three independent measurements and are presented as mean ± standard deviation (Table S2). The reproducibility of HYCOR reflects the cumulative variability of multiple experimental steps. These include the initial triplet-DNP polarization of the PAm mediator, mechanical mixing with PyA, the extent and rate of cocrystallization, relaxation losses during handling, calculation of the cocrystal polarization after accounting for residual PAm, and dissolution/transfer for solution-state detection. Thus, the standard deviations in Table S2 reflect the overall reproducibility of the current proof-of-concept HYCOR workflow, including sample preparation, cocrystallization, handling, and detection.

Section S5: Analysis of polarization signals based on the PXRD-determined composition

For an initial PyA:PAm ratio of 1:3, the composition of the HYCOR sample was determined to be PyA–PAm cocrystal and unreacted PAm in a molar ratio of 0.78:2.22 by PXRD using the internal-standard method. The hyperpolarized NMR signal intensity obtained from the HYCOR sample contains contributions from both the PyA–PAm cocrystal and unreacted PAm. The NMR contribution from unreacted PyA is negligible because its molar amount (0.005 mmol) is extremely small relative to the total sample and its signal intensity falls below the detection threshold under the present DNP conditions. To extract the net signal originating from the PyA–PAm cocrystal, the contribution from unreacted PAm was subtracted according to the PXRD-determined composition:

$$S_{\mathrm{PyA-PAm}} = S_{\mathrm{total}} - \frac{2.22}{3} \times S_{\mathrm{PAm}}$$

where $S_{\mathrm{PyA-PAm}}$ is the signal intensity arising from the PyA-PAm cocrystal, S_{total} is the total signal intensity of the HYCOR sample, and S_{PAm} is the signal intensity of PAm measured under identical DNP conditions and scaled to the corresponding molar amount. The coefficient $\frac{2.22}{3}$ represents the fraction of unreacted PAm relative to the initial PAm amount. Based on this correction, the nuclear spin polarization of the PyA–PAm cocrystal was estimated to be P (^{1}H) of 0.018% [ε (^{1}H) = 1.4 × 10^{2}] and P (^{13}C) of 0.012% [ε (^{13}C) = 3.7 × 10^{2}].

Section S6: Kinetic model for polarization loss during HYCOR

To evaluate the polarization loss during HYCOR quantitatively, we considered a simple kinetic model that combines cocrystallization kinetics with polarization relaxation during cocrystal formation. We defined

$t = 0$ as the start of mixing between the polarized PAm mediator and liquid PyA, t_{cryst} is the cocrystallization time, and t_{det} is the detection time measured from the start of mixing. The observed ^{1}H polarization at t_{det} is modeled as

$$P_{\mathrm{obs}}\,(t_{\mathrm{det}}) = P_0 \eta_{\mathrm{dist}} \eta_{\mathrm{growth}} \int_0^{t_{\mathrm{cryst}}} \frac{dX(t)}{dt} \exp\left[-\frac{t_{\mathrm{det}} - t}{T_{1,\,\mathrm{cryst}}}\right] dt.$$

Here, $P_{\mathrm{obs}}\,(t_{\mathrm{det}})$ is the detected ^{1}H polarization, P_0 is the initial ^{1}H polarization of the PAm mediator, η_{dist} is the polarization distribution factor from the initially polarized PAm protons to all protons in the PyA–PAm cocrystal, η_{growth} is the effective polarization-loss factor during cocrystal growth, $X(t)$ is the PyA-based cocrystallization fraction approximated as $X(t) = 1 - \exp\,(-k_{\mathrm{cryst}} t)$, and $T_{1,\,\mathrm{cryst}}$ is the T_1 (^{1}H) of the formed cocrystal. The factor η_{growth} includes polarization loss during transient wet solid–liquid state, molecular rearrangement, nucleation, and incorporation into the growing cocrystal before the stable PyA–PAm cocrystal is fully formed. From the PXRD-derived value $X(5\ \mathrm{s}) = 0.78$, k_{cryst} was estimated to be $0.30\ \mathrm{s}^{-1}$. The distribution factor η_{dist} was estimated to be in the range of 0.6–0.8. The lower value, 0.6, was estimated from the proton distribution in the final 1:1 PyA–PAm cocrystal. The upper value accounts for the excess PAm mediator used experimentally to accelerate cocrystallization.

Using $t_{\mathrm{cryst}} = 5\ \mathrm{s}$, $t_{\mathrm{det}} = 7\ \mathrm{s}$, $k_{\mathrm{cryst}} = 0.30\ \mathrm{s}^{-1}$, $P_0 = 0.96\%$, $P_{\mathrm{obs}}(7\ \mathrm{s}) = 0.018\%$, $T_{1,\,\mathrm{cryst}} = 9.6\ \mathrm{s}$, and $\eta_{\mathrm{dist}} = 0.6$–$0.8$, the model gives $\eta_{\mathrm{growth}} \approx 0.05$–$0.07$. The result indicates that most of the polarization loss occurs during the formation of the PyA–PAm cocrystal before the stable PyA–PAm cocrystal is fully formed.

Section S7: Dissolution triplet-DNP measurements

We used a dissolution triplet-DNP system. A superconducting magnet (SCM) of 11.7 T (Varian Inc.) was employed for the dissolution NMR experiments. We placed the 0.39 T electromagnet (EM) for triplet DNP beneath the SCM. After triplet-DNP experiments, sample in a quartz tube was manually transferred from the electromagnet to the SCM, ground into a powder at the EM, attached to a shuttle device in the stray field (~0.1 T), and then shuttled into the SCM. Chloroform was used to dissolve the polarized samples inside the NMR detection coil at 298 K.

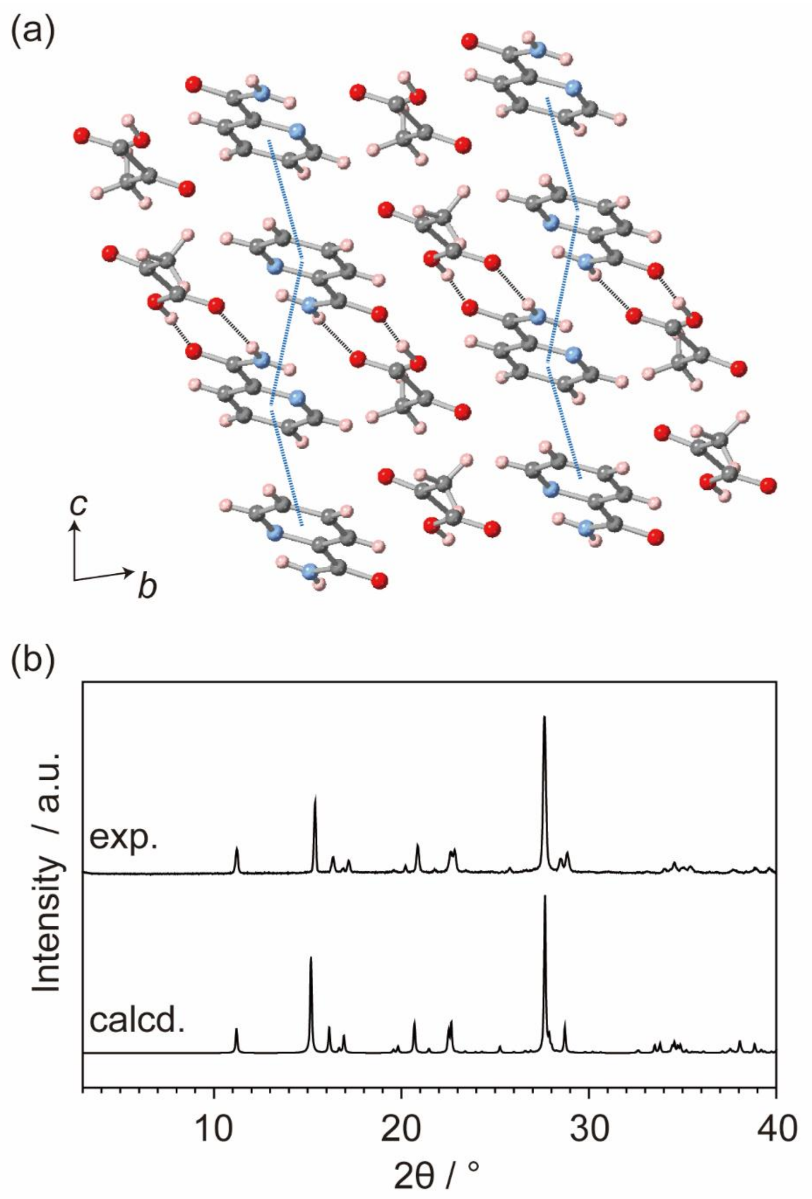

Figure S1. (a) Crystal structure of the PyA–PAm cocrystal. Acid–amide hydrogen bonds and π–π stacking interactions are depicted as black and blue dotted lines, respectively. H, O, N, and C atoms are shown in white, red, blue, and black, respectively. (b) Comparison of the experimental PXRD pattern of the PyA–PAm cocrystal (PyA:PAm = 1:1) with the calculated pattern derived from the crystal structure.

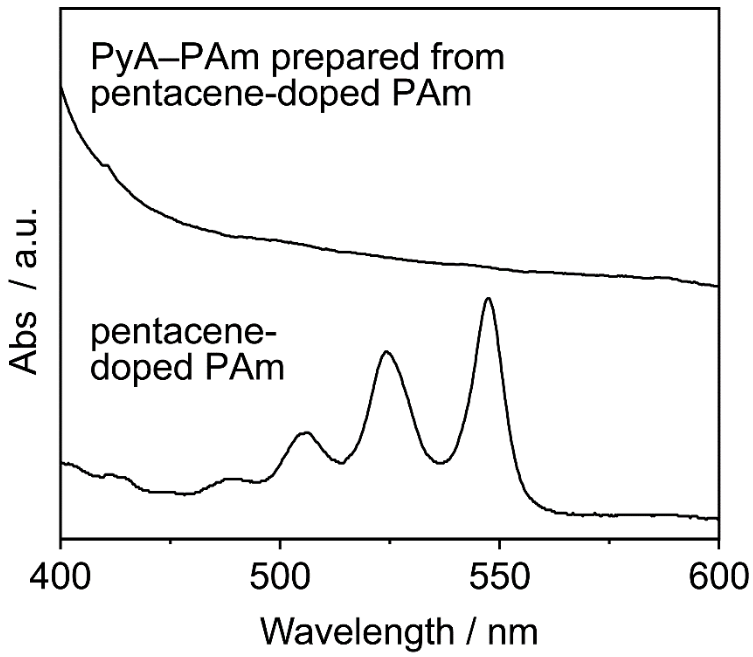

Figure S2. UV–Vis spectra of pentacene-doped PAm and PyA–PAm prepared from pentacene-doped PAm.

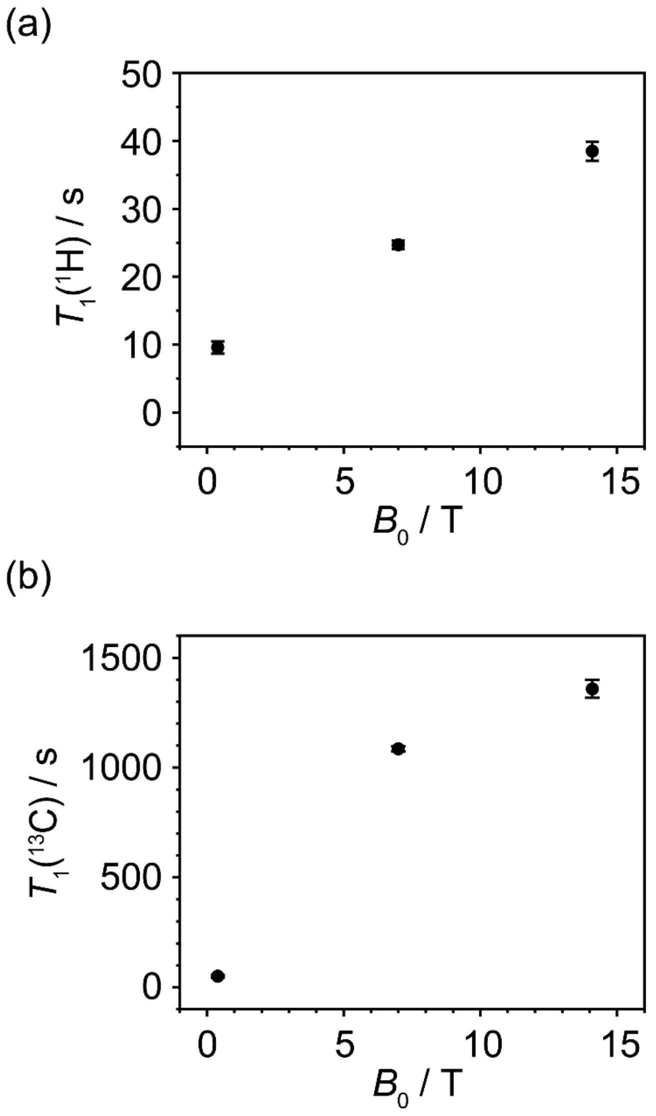

Figure S3. (a) T_1 (^{1}H) of PyA–PAm measured at 0.39, 7.0 and 14.1 T. T_1 (^{1}H) was determined by ^{1}H saturation-recovery experiments under static conditions at 0.39 T and under MAS conditions at 7.0 and 14.1 T. The resulting T_1 values are 9.6 ± 0.9 s (0.39 T), 24.7 ± 0.6 s (7.0 T) and 38.5 ± 1.4 s (14.1 T), respectively. (b) T_1 (^{13}C, COOH) of PyA–PAm measured at 0.39, 7.0 and 14.1 T. T_1 (^{13}C) at 7 and 14.1 T were measured by the Torchia CP method using ^{1}H-^{13}C CP-MAS NMR spectra after ^{1}H saturation recovery. The resulting T_1 values are 1085 ± 12 s (7.0 T) and 1358 ± 40 s (14.1 T), respectively. At 0.39 T, the T_1 (^{13}C) was estimated by fitting the signal decay of accumulated spectra obtained after the HYCOR experiment. The ^{13}C signal intensity from PyA–PAm was obtained by subtracting the contribution of unreacted PAm from the total intensity according to the PXRD-determined composition (Section S5). The resulting T_1 value is 49.9 ± 5.4 s (0.39 T). Error bars represent fitting errors from single-exponential relaxation fits.

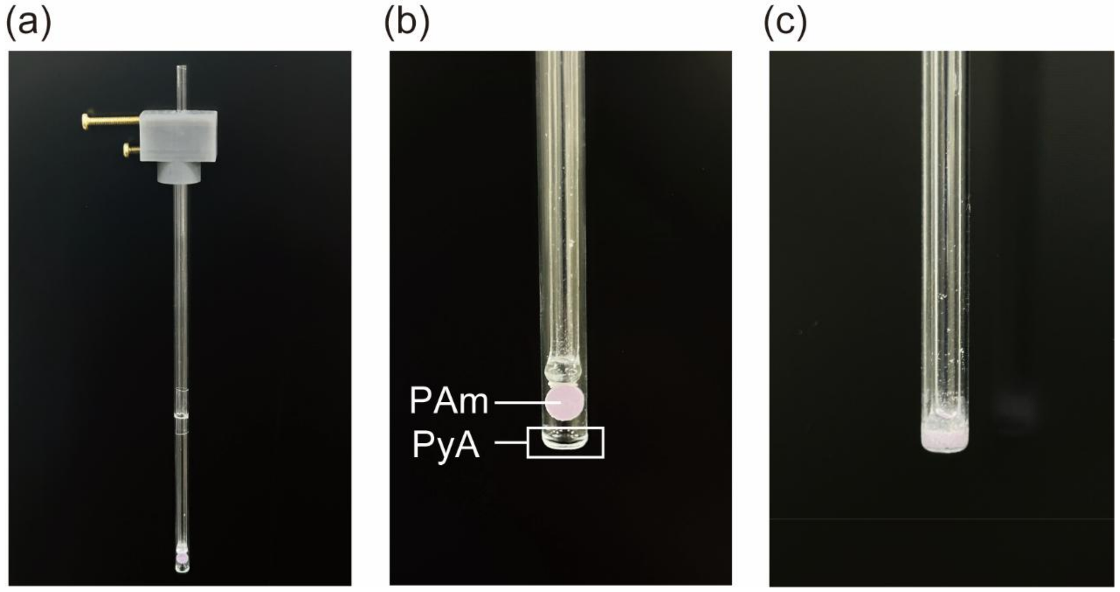

Figure S4. Photographs of the NMR sample tube for HYCOR. (a) overall view of the sample tube, (b) close-up view showing liquid PyA (bottom) and a PAm pellet (top) placed in the tube, and (c) PyA–PAm cocrystal obtained after cocrystallization.

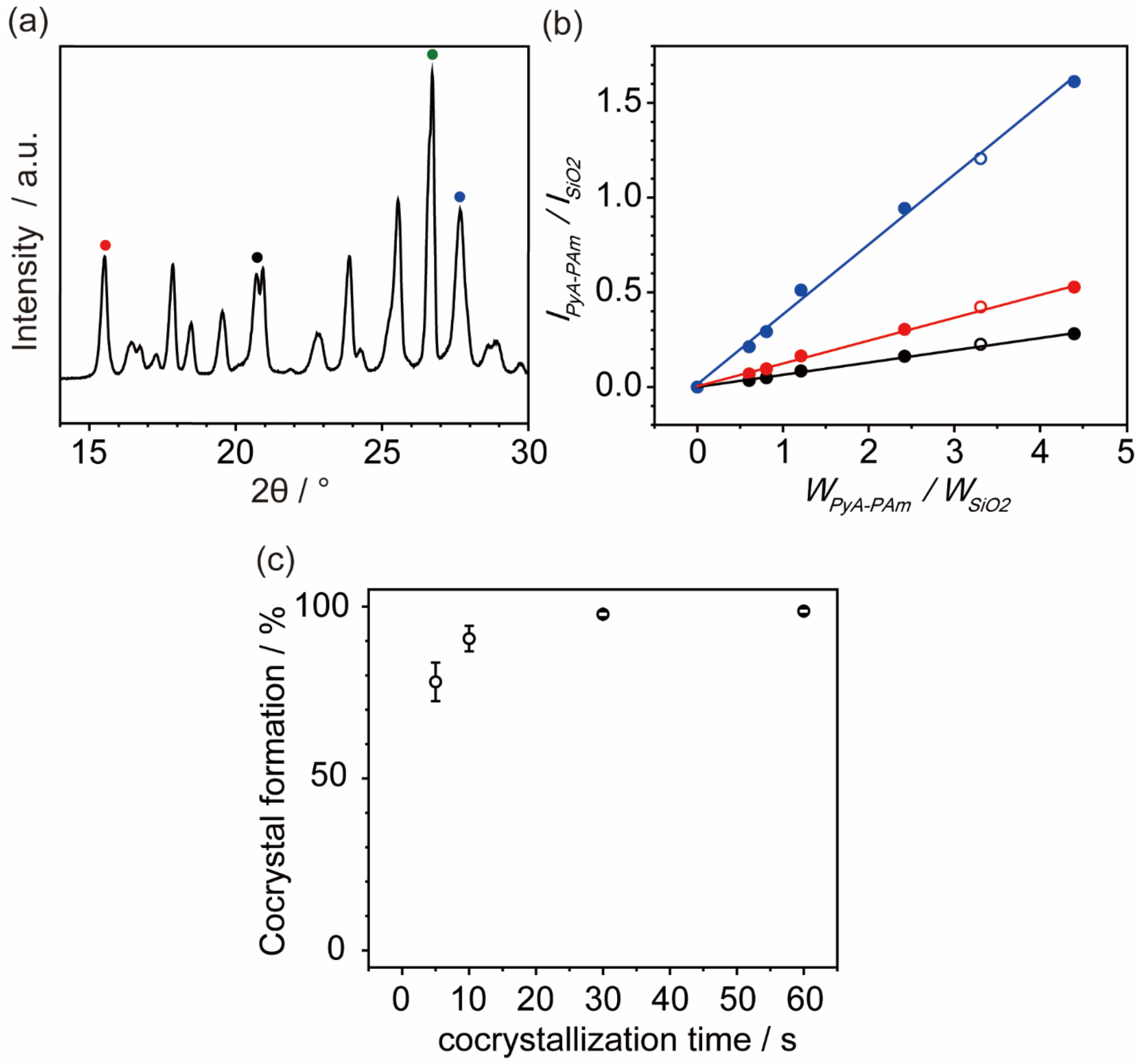

Figure S5. Quantitative PXRD analysis using the internal-standard method. (a) PXRD pattern of a mixture of the PyA–PAm-13 sample quenched at 5 s and SiO_2 for quantitative analysis. Filled circles indicate reflections used for profile fitting and peak-area integration. The SiO_2 reflection is highlighted in green, and the PyA–PAm-13 reflections are highlighted in black, red, and blue, corresponding to the datasets shown in panel (b). (b) Calibration curves constructed by plotting the integrated peak-area ratio $I_{\mathrm{PyA-PAm}}/I_{\mathrm{SiO2}}$ against the corresponding known weight ratio $W_{\mathrm{PyA-PAm}}/W_{\mathrm{SiO2}}$. Open circles represent samples quenched at the indicated reaction times by rapid cooling in liquid nitrogen. (c) Fraction of cocrystal formation (%) as a function of reaction time (5, 10, 30, and 60 s). The values represent the mean ± standard deviation of three independent measurements. The fractions at 5, 10, 30, and 60 s were 78.1 ± 5.6%, 90.7 ± 3.7%, 97.8 ± 0.9%, and 98.7 ± 0.8%, respectively.

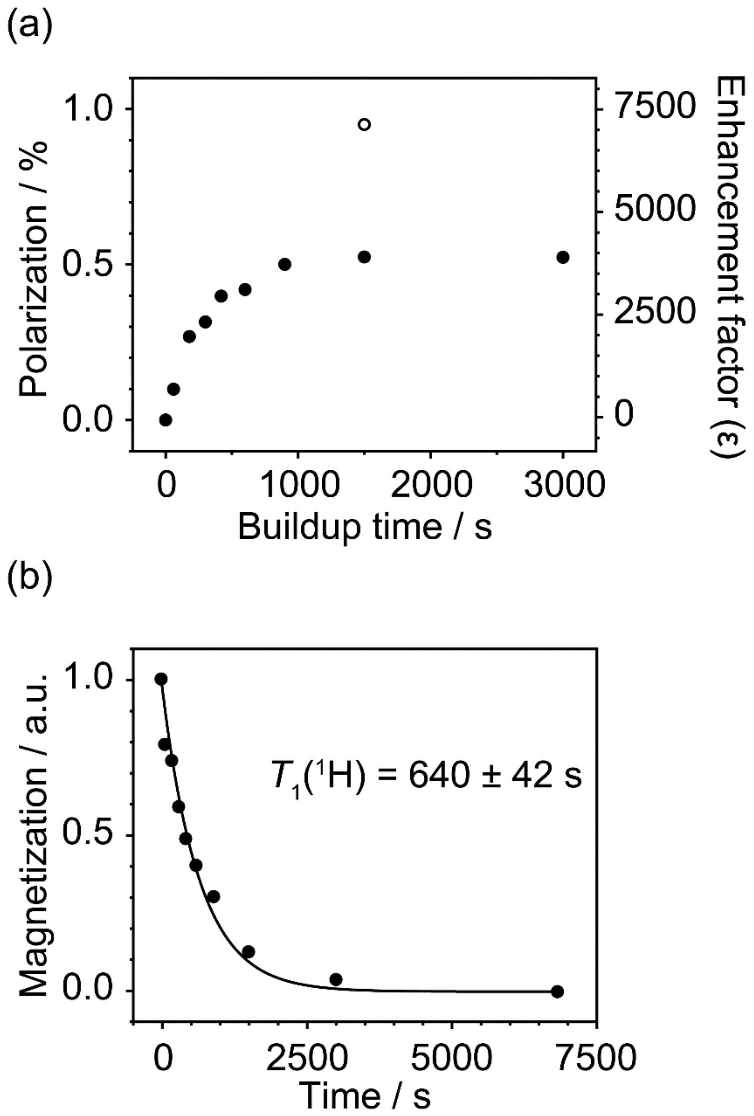

Figure S6. (a) ^{1}H polarization buildup curves of PAm doped with pentacene-d14 obtained by triplet DNP using ISE at 200 Hz under 0.39 T at 298 K. Filled circles represent the DNP buildup curve measured by repeated DNP experiments on the same sample. The open circle represents a single-shot measurement after 1500 s of DNP using a fresh sample, included to avoid the effect of sample degradation during repeated measurements. Enhancement factors were referenced to the thermal equilibrium polarization at 0.39 T using ethanol as the thermal reference. (b) T_1 (^{1}H) of the polarized magnetization of PAm, with uncertainties representing fitting errors from single-exponential relaxation fits.

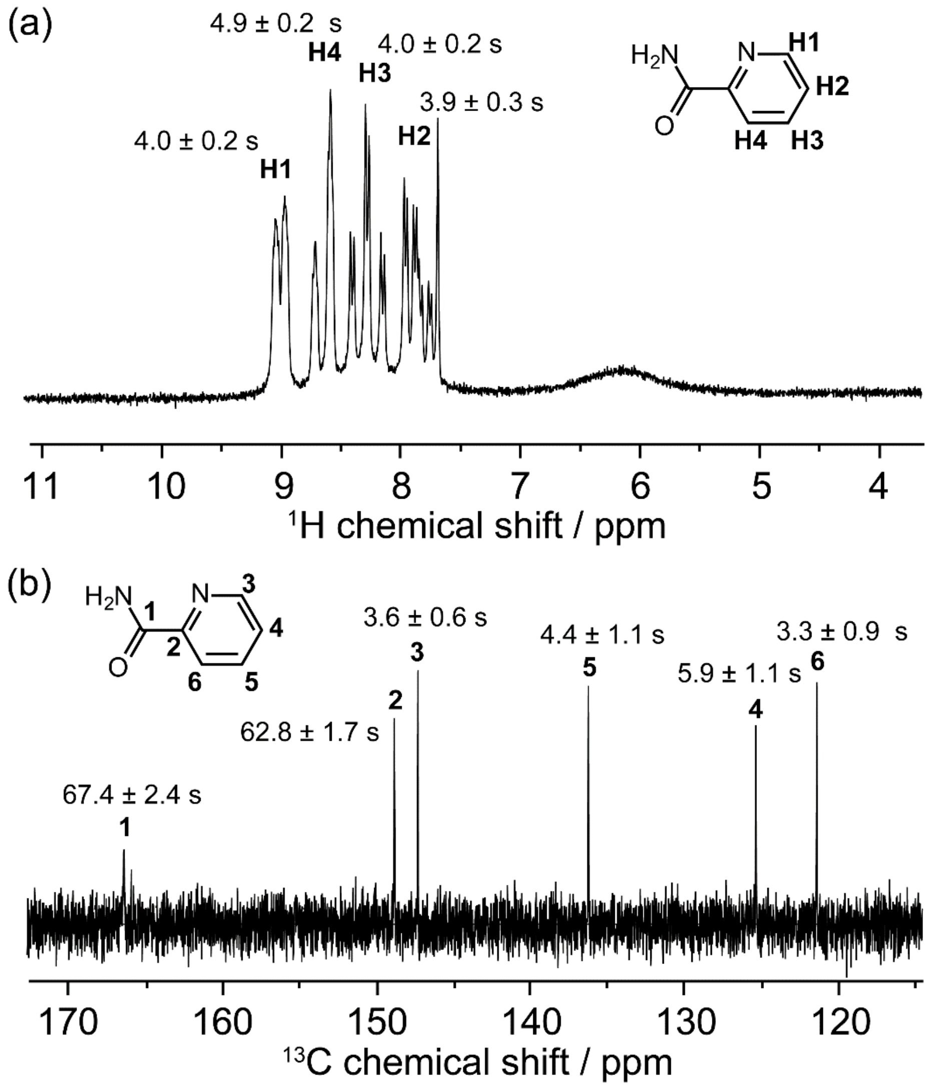

Figure S7. (a) ^{1}H and (b) ^{13}C NMR spectra of PAm in $CDCl_3$ at 1.4 T. The T_1 (^{1}H) and T_1 (^{13}C) values were obtained by the inversion-recovery method. Uncertainties represent fitting errors from single-exponential relaxation fits.

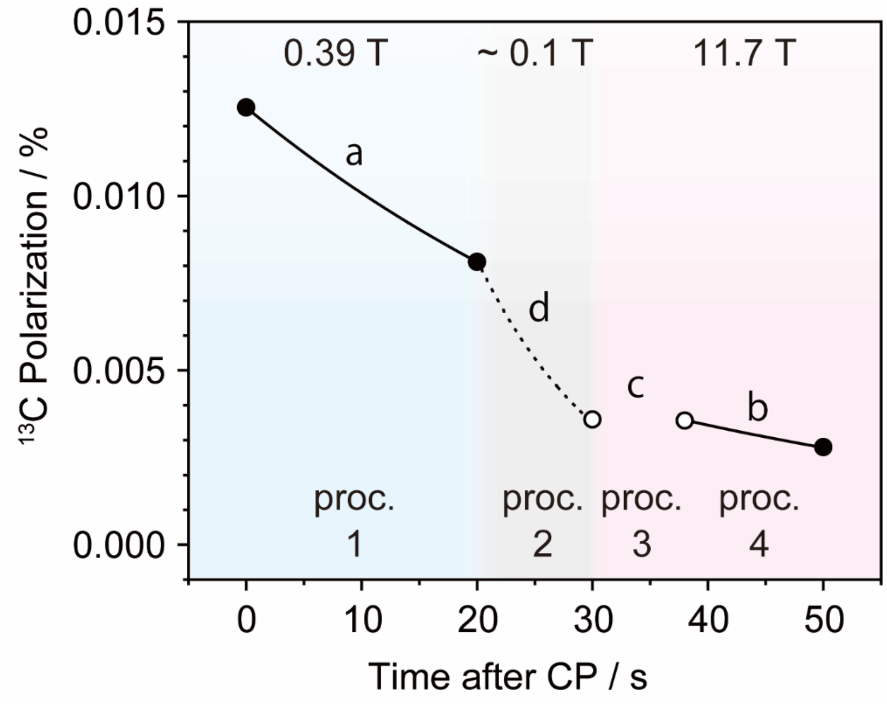

Figure S8. Decay plot of ^{13}C polarization in PyA–PAm observed after CP. The magnetic field was 0.39 T for 0–20 s (proc. 1, blue region), approximately 0.1 T for 20–30 s (proc. 2, gray region), and 11.7 T for 30–50 s (proc. 3 and 4, red region). (a) The ^{13}C polarization at 20 s (after powdering) was 0.0081%. From this value, the T_1 (^{13}C) of PyA–PAm in the solid state at 0.39 T was estimated to be 46 s, in good agreement with the independently measured T_1 (^{13}C) of 50 s (Figure S3). (b) To estimate the polarization decay at 11.7 T, the T_1 (^{13}C) of PyA in $CDCl_3$ solution was used, which was measured to be 50 s (Figure S9). The ^{13}C polarization at 38 s was back-calculated from the measured value at 50 s (0.0028%) using $P(38) = P(50) \times \exp\,(12/T_1(^{13}\mathrm{C}))$, yielding a polarization of 0.0036%. (c) The T_1 (^{13}C) of solid PyA–PAm at 11.7 T is on the order of 1000 s, indicating that the decay between 30 and 38 s is negligible. (d) The T_1 (^{13}C) of PyA–PAm in the solid state at approximately 0.1 T was estimated to be 12 s by fitting the polarization decay between 20 and 30 s using $\exp\,(-t/T_1(^{13}\mathrm{C}))$. The shorter T_1 (^{13}C) at ~ 0.1 T compared to that at 0.39 T is reasonable, given the enhanced relaxation at lower magnetic fields. Filled circles represent experimentally observed polarization values, whereas open circles indicate polarization values estimated from the corresponding T_1 relaxation times.

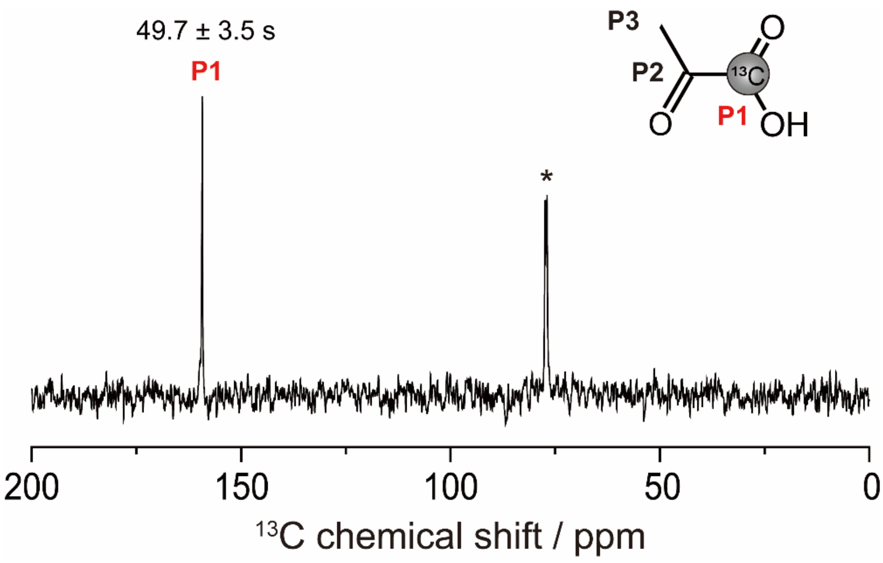

Figure S9. ^{13}C NMR spectrum of PyA in $CDCl_3$ at 11.7 T. The other ^{13}C sites in PyA (**P2** and **P3**) remain undetectable due to their low natural ^{13}C abundance and weak signal intensity. The T_1 (^{13}C) value of **P1** was measured by the inversion-recovery method. The asterisk denotes the signal arising from chloroform. Uncertainties represent fitting errors from single-exponential relaxation fits.

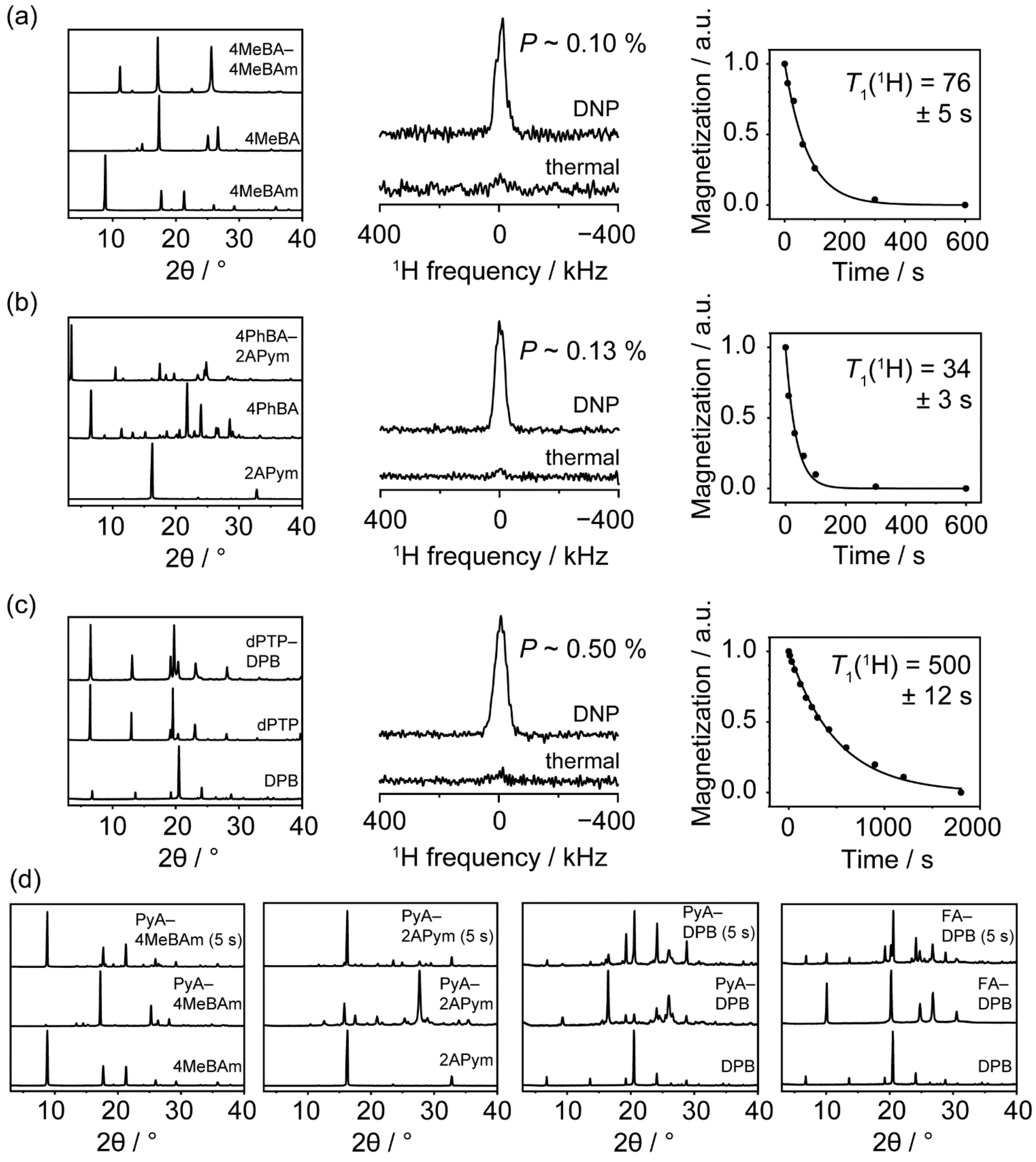

Figure S10. (a–c) PXRD patterns, triplet-DNP-enhanced and thermal ^{1}H NMR spectra, and T_1 relaxation curves of (a) 4MeBA–4MeBAm (4-methylbenzoic acid–4-methylbenzamide), (b) 4PhBA–2APym (4-phenylbenzoic acid–2-aminopyrimidine), and (c) dPTP–DPB (d_{14}-*p*-terphenyl–1,4-di(4-pyridyl)benzene) cocrystals. (d) PXRD patterns after rapid mixing of PyA (pyruvic acid) or FA (formic acid) with the cocrystal mediators (upper: carboxylic acid/mediator = 1:3, ground for 5 s; middle: carboxylic acid/mediator = 1:1, ground for 10 min; lower: mediator cocrystal).

Table S1. Crystallographic data for PyA–PAm.

Compound	PyA–PAm
Temperature	293 K
Chemical formula	$C_9H_{10}N_2O_4$
Space Group	*P*-1
a / Å	7.6245(3)
b / Å	8.1837(4)
c / Å	15.7155(8)
α / °	80.516(4)
β / °	82.033(4)
γ / °	85.227(4)
Volume / Å^3	956.02(8)
Z	2
R	0.0979
wR_2	0.2458
GOF	1.118

Table S2. ^1H and ^{13}C polarization ratios, P (^1H) and P (^{13}C), and the corresponding enhancement factors ε for PyA–PAm. For ^1H, solid-state enhancement factors were calculated relative to the thermal equilibrium polarization at 0.39 T using ethanol as the thermal reference. For ^{13}C, the frequency-matched ^1H signal of ethanol measured at 0.10 T was used as the thermal reference. The enhancement factor for the dissolved sample was calculated relative to the thermal equilibrium polarization at 11.7 T. Polarization values are presented as mean ± standard deviation from three independent measurements.

Stage	Sample	P (^1H)	ε (^1H)	P (^{13}C)	ε (^{13}C)
mediator (solid)	PAm	0.96 ± 0.08	7200 ± 600	0.61 ± 0.02	18000 ± 700
mixture (solid, 5 s synthesis)	PyA–PAm + unreacted PAm and PyA	0.57 ± 0.02	4300 ± 200	0.074 ±0.002	2200 ±70
cocrystal (solid)	PyA–PAm	0.018 ± 0.003	140 ± 20	0.012 ± 0.003	370 ± 90
dissolved (liquid)	PyA	–	–	0.0028 ± 0.0006	2.8 ± 0.6

References

(1) Alexander, L.; Klug, H. P. Basic Aspects of X-Ray Absorption in Quantitative Diffraction Analysis of Powder Mixtures. *Anal. Chem.* **1948**, *20*, 886–889.
(2) Lou, Y.; Zuo, L. Quantification of Losartan Potassium Polymorphs Using Powder X-Ray Diffraction. *J. AOAC Int.* **2021**, *104*, 579–584.
(3) Takeda, K. OPENCORE NMR: Open-source core modules for implementing an integrated FPGA-based NMR spectrometer. *J. Magn. Reson.* **2008**, *192*, 218–229.
(4) Takegoshi, K.; McDowell, C. A. A "magic echo" pulse sequence for the high-resolution NMR spectra of abundant spins in solids. *Chem. Phys. Lett.* **1985**, *116*, 100–104.
(5) Henstra, A.; Lin, T. S.; Schmidt, J.; Wenckebach, W. T. High dynamic nuclear polarization at room temperature. *Chem. Phys. Lett.* **1990**, *165*, 6–10.
